# Communicable Diseases Surveillance System in East Azerbaijan Earthquake: Strengths and Weaknesses

**DOI:** 10.1371/currents.dis.9085e38035f25b34f093f357ac2c3973

**Published:** 2014-12-08

**Authors:** Javad Babaie, Farin Fatemi, Ali Ardalan, Hamed Mohammadi, Mahmood Soroush

**Affiliations:** Department of Disaster Public Health, School of Public Health, Tehran University of Medical Sciences, Tehran, Iran; Department of Disaster and Emergency Health, National Institute of Health Research, Tehran University of Medical Sciences, Tehran, Iran; Department of Disaster Public Health, School of Public Health, Tehran University of Medical Sciences, Tehran, Iran; Department of Disaster & Emergency Health, Iran's National Institute of Health Research; Department of Disaster Public Health, School of Public Health, Tehran University of Medical Sciences, Tehran, Iran; Harvard Humanitarian Initiative, Harvard University, Cambridge, Massachusetts, USA; Department of Disaster Public Health, School of Public Health, Tehran University of Medical Sciences, Tehran, Iran; Communicable Diseases Surveillance Department,Ministry of Health, Treatment and Medical Education, Tehran, Iran

**Keywords:** communicable diseases, disaster, surveillance system

## Abstract

Background: A Surveillance System was established for 19 diseases/syndromes in order to prevent and control communicable diseases after 2012 East Azerbaijan earthquakes. This study was conducted to investigate the strengths and weaknesses of the established SS. Methods: This study was carried out on an interview-based qualitative study using content analysis in 2012. Data was collected by semi-structured deep interviews and surveillance data. Fifteen interviews were conducted with experts and health system managers who were engaged in implementing the communicable disease surveillance system in the affected areas. The selection of participants was purposeful. Data saturation supported the sample size. The collected data was analyzed using the principles suggested by Strauss and Corbin. Results: Establishment of the disease surveillance system was rapid and inexpensive. It collected the required data fast. It also increased confidence in health authorities that the diseases would be under control in earthquake-stricken regions. Non estimated denominator for calculating the rates (incidence & prevalence), non-participation of the private sector and hospitals, rapid turnover of health staff and unfamiliarity with the definitions of the diseases were the weak points of the established disease SS. Conclusion: During the time when surveillance system was active, no significant outbreak of communicable diseases was reported. However, the surveillance system had some weaknesses. Thus, considering Iran’s susceptibility to various natural hazards, repeated exercises should be conducted in the preparedness phase to decrease the weaknesses. In addition, other types of surveillance system such as web-based or mobile-based systems should be piloted in disaster situations for future.

## Introduction

Two earthquakes with magnitudes of 6.3 and 6.4 in the Richter scale shook wide areas of East Azerbaijan in the northwest of Iran, particularly Ahar, Heris, and Varzaghan counties on Aug 11, 2012 1 . The earthquakes killed 228 persons and injured more than 3000 2 .

Disasters in the societies are usually followed by different adverse outcomes such as death, loss, physical and psychological injuries, outbreak of infectious diseases, extensive movements of population, disruption of vital services, and destruction of infrastructures^3^ . On the other hand, destruction of the houses leads to temporary settlement of homeless people and inaccessibility or inadequate access of the population to health facilities and services^4^^,^ 5 .

The East Azerbaijan earthquakes confirmed the authenticity of mentioned points once more. The newly-established hospital of Varzaghan, the second floor of Bagherololum Hospital in Ahar, and Imam Hossein Hospital in Haris were damaged and evacuated. Eighty eight rural health houses^1^ and 10 rural health centers were damaged, as well. Although none of the health workers were injured, due to the difficult mental conditions, they were not able to render services. Thus, the routine health systems in the earthquake-stricken areas were disordered.

Such conditions are more vulnerable to outbreaks of communicable diseases (CD). Several outbreaks of measles in refugees’ camps^6^ , and the outbreak of diarrheal diseases in Texas following the Katrina, Bangladesh flood, earthquake of Haiti^7^ are examples of CD outbreaks after disasters.

Therefore, CD management is the most important component of the health system response to disasters and the surveillance system (SS) is its critical division. Hence, health systems commonly try to set up a system for the surveillance of CD very soon after the disaster. For instance, such surveillance was established following the Hurricane Katrina in Louisiana and New Orleans, the 2010 Haiti earthquake, the 2010 Pakistan floods^8^^,^ 9 , the 2008 Sichuan earthquake^10^ , the 2011 Japan earthquake and tsunami^11^ , and Bam and Zarand earthquakes^12^^,^ 13 .

In this earthquake, the health center of East Azerbaijan in collaboration with the Ministry of Health designed and implemented a communicable diseases surveillance system in the affected areas. Although disasters are terrible events, they can be very informative and learning from them can reduce their adverse effects in future disasters. Therefore, response programs and the performed actions must be carefully assessed and their pitfalls should be extracted. We can provide an effective response by avoiding previous challenges and using strengths. Therefore, this article focuses on the disease surveillance system in East Azerbaijan earthquake and its strengths and weaknesses.

## Methods

This study was carried out in 2012. We used content analysis as a research method for the subjective interpretation of the interview data through a systematic classification process of coding and identifying concepts. We conducted fifteen interviews with experts and health system managers with previous coordination which were held in their official rooms in a quiet environment. The selection of participants was purposeful and all of them were involved in the established surveillance system in the earthquake-stricken areas. Table 1 highlights the demographic features of the study participants.

**Table 1: The demographic characteristics of the experts and managers participating in interviews:** 

Gender (%)	Male	100%
Job experience	Mean	18.6 (Year)
Field of knowledge (n)	Medical science	8(53.3%)
	Public health	4(26.6%)
	Health management	3(20.1%)
Level of education (n)	General practitioner	8(53.3%)
	Master of science	3(20.1%)
	Bachelor of science	4(26.6%)
Level of work (n)	Province	7(46.6%)
	County	5(33.3%)
	Health center	1(6.66%)
	Health team	2(13.33%)

**Flow of information and reports of the disease surveillance after 2012East Azerbaijan earthquake.**
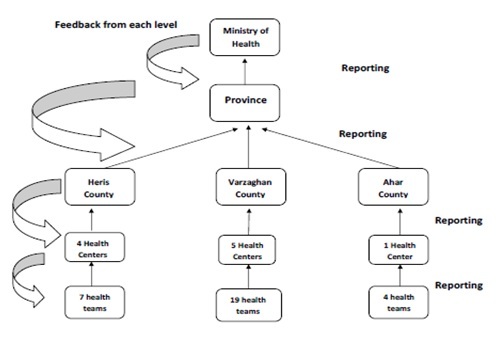

Data saturation supported the sample size and the interviews were continued until saturation of each concept was achieved. Each interview lasted between 35 to 60 minutes (mean: 48 minutes). The interviews were conducted in local language (Turkish) and transcribed by the same interviewer for analysis.

The interviews were deep and semi-structured. We used an interview guide that included a list of general questions (see appendix).

Depending on the responses to initial questions, additional clarifying questions were asked for better clearance during interviews whenever needed. The data were analyzed manually following the principals set forward by Strauss and Corbin^15^ . Data collection and data analysis took place simultaneously in order to identify ideas, which then guided the next interview.

During the open coding phase, all the interviews were read several times and key words were determined in the text. Primary codes were extracted. After that, codes and data were compared for similarities and differences. Categories and sub-categories were developed. In conformity with the methodology of content analysis^15^ , they were all performed by the same investigator for all interviews. Data validation was carried out by member check^16^ . During this process, the transcriptions and a summary of primary results (codes and categories) were checked by the participants in order to improve trustworthiness.

Eleven categories were extracted from interviews analysis. Four and seven categories were classified as strengths and weaknesses.

We also investigated documents and regularly collected data by the surveillance system during September to calculate the trend of the prevalence of the CD.

## Description of Established Surveillance System

Three days after the earthquakes in East Azerbaijan, the surveillance system of CD was designed for detecting and monitoring 19 diseases/syndromes in the affected areas. Thirty health teams in 10 health centers were involved in establishing the SS. All health teams had general physicians, obstetricians/nurses, environmental health and disease control technicians/specialists.

Communications and reports were two-sided, meaning that the data was collected from health teams, and concluded and analyzed in the health centers and districts level. Then, the report was sent to the health center of the province and finally to the highest level of the Ministry of Health. During this process, feedback was met from each layer and this trend was repeated on a daily basis. All the data was recorded manually by health teams in the National Emergency Operation Plan^17^ (E.O.P) forms.

Nineteen diseases/syndromes that required surveillance in this system were diarrhea, dysentery, water and food-borne outbreaks, animal bites, snake and scorpion bites, botulism, acute respiratory infection, influenza-like illness (ILI), anthrax, pertussis, meningitis, cutaneous leishmaniasis, malaria, tuberculosis, acute flaccid paralysis, sexually transmitted disease, contamination by louse, acute jaundice, and other cases.

## Results

***Surveillance Data***

The surveillance duration was divided to two phase. The first phased started from Aug 15 to September 21, 2012, and the second phase from September 22 to December 20, 2012. Within the first 40 days after the earthquake (until September 21, 2012), the reported diseases were watery diarrhea (1332 cases), acute respiratory infection (ARI) (1156 cases), influenza-like illness (ILI) (164 cases) and animal bite (56 cases). In the second phase (from September 22 until December 20), the majority of the diseases were 206 cases of acute watery diarrhea, 95 cases of influenza-like illness, and 92 cases of acute respiratory infections. Moreover, 5 cases of limited food-borne outbreak were diagnosed and certified within a 4-month surveillance period, which were stopped immediately by undertaking controlling measures. There was no specific problem in terms of epidemics in the earthquake-stricken areas, which made authorities and people confident that the diseases were under control.

***Interview data***

**Strengths**

***1- Rapid establishment:*** The mentioned surveillance system was operational within three days after the earthquake. It could provide the officials and decision makers with the required information for the management of communicable diseases in affected areas. One of the participants in this study stated: *“It was the third day that experts came from the Ministry of Health. We collaborated with each other and commissioned this system. Because we did not know what would happen, we were worried about the future of the region, but this system gave us the information and data we required day after day.”*

**Incidence of communicable diseases in East Azerbaijan earthquake stricken areas, 23 Aug-22 Sep,2012:**
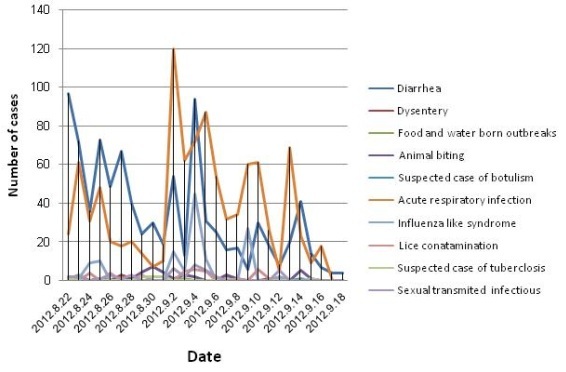

***2- No need for additional facilities and resources:*** The system was built based on the available facilities. No additional resources were employed for its establishment that could incur costs upon the health system. Furthermore, the system experts had prior familiarity with its execution process. One of experts said: *“In fact, we used available facilities and caused no other extra costs. Everything was available from the past. We only explained the procedure to the personnel and distributed the forms. The data was collected and sent by teams. It caused no extra expenses for us.”*

***3- Capability of quick data collection*** **:** This system collected and transferred the data related to diseases quickly so that the information was supplied to the responders at most within one day. One participant said*: “Our information was almost up-to-date. We were completely aware of the region with a one-day delay. If there was a concern, they informed us immediately by phone. In general, the disease status in the area was under control.”*

***4- Increase confidence in the health system:*** This system made officials confident about controlling the health status of earthquake-stricken areas. Thus, they were assured there was no outbreak of communicable diseases. Moreover, data related to the occurrence and incidence of communicable diseases indicated that performed actions were appropriate and efficient. One of the physicians stated: *“For the first time, we faced such conditions. Since there was much destruction and it was in the summer, we were concerned about the prevalence of diarrheal diseases but day after day, we became certain that the conditions were controlled well.”* Another expert said: *“At the beginning, there were rumors about some diseases in the region, but this system ended these rumors upon providing timely information. The officials confidently declared that there was no concerning case.”*

**Weaknesses**

***1-Non-estimated denominator:*** The region population was not specified. A lot of people from other cities were present in the damaged villages and the region population changed every day. Consequently, the rates (prevalence and incidence rates) could not be calculated. Only the raw statistics of diseases were used and the trend of the diseases was controlled based on number. One participant in this study said: *“The population changed every day. A lot of people came from other places and resided in the villages. Plenty of these villagers immigrated to Tabriz. Due to the current status of the region, they returned for a lot of reasons and then resided in the villages. We truly did not know how many people resided in the region.*” Another expert said: *“We didn’t know to apply which number in the denominator. We were obliged to apply the number of all daily visits, but it was not a correct number and depended on the number of visits which health teams paid on that day. If the number of visits was high, the statistics of our diseases increased as well. If there were fewer visits, the number of patients decreased, too. Daily statistics were not comparable at all. We knew it, but didn’t know what to do.”*

***2-Non-participation of the private sector and general hospitals:*** In this SS, the data was collected and sent only by health teams which were dispatched to the region by the province health center. The private sector and public hospitals available in the region did not participate in the SS. Therefore, the diseases statistics only included part of the clients who were consulted by the health team and no statistics of total patients existed in the region.

One of the participants stated: *“We collected daily statistics only from our teams and didn’t intervene in clinics and hospitals. If the disease was reportable according to the WHO, they informed us, but we have received no report yet.”*

***3- Non-participation of the staff of health houses*** **:** Despite activating the health houses and establishing health sites in all villages with a population over 20 households, the implemented surveillance system did not use the health staff for data collection and their relationship with the system was not defined although they were relatively familiar with the disease reporting system. One of the experts said: *“We didn’t know at all how to communicate with the region’s rural health houses staff. They also didn’t know how and what to report. It is not also clear now.”*

***4- Lack of inter-sectoral collaboration:*** The established surveillance system had no communication with other health sites in the earthquake-stricken regions and no effort was observed to communicate with them or attract their participation. For example there were some other relief teams from Red Crescent that provided health care. But their patients didn’t include the SS. Consequently our data about the disease incidence and prevalence was incomplete. One of the participants said: *“We didn’t know how to communicate with health sites at all. They also didn’t know how and what to report. It is not also clear now.”*

***5- Rapid turnover of health staff:*** Physicians and members of the health centers came from other cities of the province and were usually present in the area for three days before replacing by another team. The new health teams were not familiar with data collection and reporting procedures in spite of brief training on the communicable diseases SS, definitions of diseases/syndromes and daily report process before starting their task. It lasted at least one day to become acquainted with the tasks. As a result, the information continuity was disturbed and new and repeated cases created problems in patient classification. One of the interviewees said: *“Almost every day, we had a new team. It took one day to brief them, and the data on that day was not useful any more. It was our problem and we were compelled to modify their data which was not correct.” *****

***6-Poor adherence by physicians on agreed case definition:*** When health teams entered the region and before they undertook their daily tasks, the definitions of under surveillance diseases were explained to them but in practice, some physicians categorized and reported the diseases based on their former information which was different from standard definitions used in the SS. Some physicians did not also accept the definitions at all. They believed that these definitions were not acceptable academically. One of the experts stated his opinion as below: *“Someday, I noticed that one of the health teams reported 17 cases of influenza-like illness (ILI) whilst we didn’t have any cases until then. I was astonished and concerned, and I followed up the report immediately and realized that the problem was the definition of the disease and they were not really influenza-like illness (ILI). Such cases were intensively observed in animal bite cases.”*

***7- Inconsistency of data collection tools:*** After the surveillance system was implemented, data collection forms changed. As a result, the collected data was different from previous data and the trend of some under supervision diseases/syndromes was not comparable to the previous trends anymore. This variety in the forms hindered the trend of disease control within the next days in practice. One of the interviewees said: *“We changed our forms on September 22^nd^. After that, we set aside the daily disease surveillance. We saw the reports but the data was not comparable to previous data anymore.”*

## Discussion

Following the twin earthquakes in the northwest of Iran in East Azerbaijan, a surveillance system was implemented by governmental health organization for 19 communicable diseases/syndromes with a minimal delay. One of the most important strong points of the implemented system was the familiarity of the health staff with it. Quick implementation with minimum costs was another advantage of the established SS. Nevertheless, some weaknesses were recognized about the surveillance system such as non estimated denominator, non-participation of the private sector and general hospitals, non-participation of the staff of health houses, lack of inter-sectoral collaboration, and inconsistency of data collection tools.

The established surveillance system could successfully control the region’s health condition and lack of no serious epidemics confirmed its success and effectiveness. The surveillance system was designed and implemented by the Ministry of Health in collaboration with other subsidiary levels whilst in the 2010 Haiti earthquake^18^ , international organizations such as PAHO, the USA Diseases Management Center (CDC), etc also participated in establishing the SS. However, it should be considered that the intensity and extent of the affected areas and people in the Haiti earthquake^18^ was much more than the East Azerbaijan earthquake.

The most common diseases in this earthquake were watery diarrhea, acute respiratory infections, influenza-like illness and animal bite. While in the 2010 Haiti earthquake, acute respiratory infections and suspected cases of malaria and fever due to unknown causes had higher incidence rates^18^^,^ 23 . In 2010 Pakistan floods, however, skin diseases, acute respiratory infections, and acute diarrhea were the most frequent diseases^10^ and during Hurricane Katrina, influenza-like syndrome showed the highest incident rate^9^ . Although due to the deviation in the diseases/syndromes under surveillance, the precise comparison of common diseases is not possible major common diseases in all investigated disasters included acute respiratory infections, acute diarrheas and influenza-like syndrome^9^ . The speed of setting up the communicable diseases surveillance system in the East Azerbaijan earthquakes was very high and it was operational within only 3 days after the earthquakes. In the Haiti earthquake, the designed surveillance system was implemented after over 2 weeks in the affected areas^23^ .

Another advantage of this surveillance system was its adaptation with the current surveillance system in Iran. Therefore, familiarity with this system helped involved health staff to act more skillfully and efficiently. Data was collected manually and reported daily according to the registry system of patient referral. This manual registry system could have human bias. In a study about Hurricane Katrina, a syndromic surveillance system was implemented utilizing a web-based data bank and the data of the diseases was recorded daily from existing population centers in the site. Although the web-based surveillance system has some advantages including increased coverage, accurate and timely collected data and offering regularly feedback, the main shortage of the web-bases surveillance system is disruption of telecommunication infrastructures and damaging computers. In Sichuan earthquake in China, the web-based surveillance system disabled. Thus, a mobile-based surveillance system was developed for monitoring communicable diseases^20^^,^ 28 . In 2010 Pakistan floods, the “Diseases Quick Warning System”, which was available from the past, was strengthened^10^ .

In the Iranian system, 19 health threatening diseases/syndromes were monitored by 30 health teams while in the established surveillance system in the Haiti earthquake, 25 health conditions were monitored by 51 clinics and hospitals^18^ . In 2010 Pakistan floods, 13 diseases and syndromes were monitored. Different types and number of diseases were considered for surveillance in the past disasters. These differences seem to be due to executive capabilities of the affected countries and health conditions in the disaster-stricken areas. The CD incidence in the Azerbaijan earthquake had a descending trend similar to past disasters (USA, Pakistan, Haiti, etc.). The major problem of implementing the surveillance system was related to the lack of agreement on case definitions for monitoring diseases among physicians. This problem was observed in pervious disasters, as well. Challenges of the surveillance system in the Pakistan floods were non-application of standard definitions for disease cases, consequent differences in the reports, lack of acceptability of designed forms, and lack of data analysis in the cities. More attention should be paid to training the individuals who are supposed to be involved in visiting patients at the start of the surveillance system.

Excessive focusing on the daily reports wasted the resources. Several studies have also confirmed this finding^8^^,^ 22 ^,^ 24 ^,^ 29 . On the other hand, in this system, outpatient diseases received more attention that has a higher sensitivity and lower specificity^9^ .

Lack of coordination among the cities in implementing the disease surveillance system caused several water and food borne outbreaks , but the results showed that the established surveillance system in East Azerbaijan was successful in timely detection and control of the monitored diseases. Likewise, the result of another study showed the challenges of the surveillance system for homeless people after the Haiti earthquake. Communication and coordination difficulties between public organizations, limitations in using data, incomplete reports, and the multiplicity of reporting organizations were some examples of these challenges^9^^,^ 30 .

**Conclusion**

The established surveillance system functioned well in controlling CDs in the earthquake-stricken regions of East Azerbaijan. Because, no significant outbreak was reported until the end of December 2012 (when the surveillance system was active) although it was summer and the affected regions were susceptible to outbreaks of CD. However, there were some weaknesses in implementing and developing the SS.

Considering the susceptibility of Iran to various natural hazards, designing a web-based surveillance system for recording and collecting data is essential at the time of disaster.

The authors also suggest that repeated exercises should be associated with this new system to validate the mortality estimations and acquainting experts with the performance of the system. Periodic censuses ensure that an accurate denominator is used to obtain rates in order to prevent bias estimations and increase the acceptability of surveillance system in the community.

## Footnotes

[1] -Islamic Republic of Iran has a unique health system in EMRO. It is divided to three levels. The first level includes Health Houses and Rural Health Centers in rural areas. A Health House is set up in a village or a group of villages which is responsible for providing PHC for 500-2500 rural population. There is a Rural Health Center for every 4-7 Health Houses. They have general physicians and also health technicians. The staff of the Health House is trained for two years and is called "Behvarz"^26^ . In urban areas, Health Posts and Urban Health Centers play the role of Health Houses and Rural Health Centers. Health Posts are staffed by technicians. The Districts Health Centers and General Hospitals are in the second level. This level is responsible for having supervision on the first level of the health system and also providing more special treatment for referral patients. The third level includes health deputies of medical universities, and educational and specialty hospitals^27^^,^ 26 .

## Corresponding author

Ali Ardalan, MD, PhD. 78, Italia Ave, Department of Disaster Public Health, School of Public Health, Tehran University of Medical Sciences, Tehran, Iran. E-mail: aardalan@tums.ac.ir

## Appendix 1

### Interview Guide

Interviewees name and family:                                       

Education:

Interview Date:

Starting Time:

Ending Time:

General Questions

1. How was the process of establishing the disease surveillance system?
2. How is the operational trend of this surveillance system in collecting data?
3. What is your idea about the strong points of the established surveillance system?
4. What is your idea about the weak points of the established surveillance system?

Depending on responses to initial questions, additional clarifying questions will be asked for better clearance during interviews whenever needed.
